# Flagellin-based electrochemical sensing layer for arsenic detection in water

**DOI:** 10.1038/s41598-021-83053-y

**Published:** 2021-02-10

**Authors:** Hajnalka Jankovics, Patrik Szekér, Éva Tóth, Balázs Kakasi, Zoltán Lábadi, András Saftics, Benjamin Kalas, Miklós Fried, Péter Petrik, Ferenc Vonderviszt

**Affiliations:** 1 Research Institute of Biomolecular and Chemical Engineering, University of Pannonia, P.O. Box 125, Veszprém, 8200 Hungary; 2 Institute of Technical Physics and Materials Science, Centre for Energy Research, P.O. Box 49, Budapest, 1525 Hungary; 3 Institute of Microelectronics and Technology, Óbuda University, P.O. Box 112, Budapest, 1431 Hungary

**Keywords:** Molecular engineering, Nanobiotechnology, Nanobiotechnology, Nanoscale materials, Electrochemistry

## Abstract

Regular monitoring of arsenic concentrations in water sources is essential due to the severe health effects. Our goal was to develop a rapidly responding, sensitive and stable sensing layer for the detection of arsenic. We have designed flagellin-based arsenic binding proteins capable of forming stable filament structures with high surface binding site densities. The D3 domain of *Salmonella typhimurium* flagellin was replaced with an arsenic-binding peptide motif of different bacterial ArsR transcriptional repressor factors. We have shown that the fusion proteins developed retain their polymerization ability and have thermal stability similar to that of wild-type filament. The strong arsenic binding capacity of the monomeric proteins was confirmed by isothermal titration calorimetry (ITC), and dissociation constants (K_d_) of a few hundred nM were obtained for all three variants. As-binding fibers were immobilized on the surface of a gold electrode and used as a working electrode in cyclic voltammetry (CV) experiments to detect inorganic arsenic near the maximum allowable concentration (MAC) level. Based on these results, it can be concluded that the stable arsenic-binding flagellin variant can be used as a rapidly responding, sensitive, but simple sensing layer in a field device for the MAC-level detection of arsenic in natural waters.

## Introduction

We have recently reported the development of protein-based sensing layers for the detection of Ni(II) in water. In that work, various Ni-binding flagellin variants were constructed by mutations in the D3 domain or replacement with a histidine-rich polypeptide motif. We demonstrated that by immobilizing nanotubes built from mutant flagellins by in vitro polymerization on the surface of biochips, a Ni(II) ion concentration of 1 μM could be detected, which is close to the maximum allowable concentration (0.34 μM)^1^. These results prompted us to create novel flagellin-based sensor layers that, when incorporated into a biosensor, can be suitable for monitoring arsenic pollution in natural waters.

Arsenic is a naturally occurring toxic metalloid, distributed everywhere on Earth: it enters the environment from both geological and human sources^2,3^. Its presence is strongly associated with several health problems, including cancer, neurological and cardiovascular defects^4^. Health threshold limit in drinking water, specified by the World Health Organization (WHO), is 10 µg/L (equivalent to 10 ppb or 0.13 µM). Precise determination of such a low concentration in a stationary laboratory environment can be performed using sophisticated instrumentation (like inductively coupled plasma mass spectrometry, atomic absorption spectroscopy or liquid chromatography tandem mass spectrometry), which are inherently not suitable for field testing. On the other hand, due to the toxicity and ubiquity of arsenic, development of sensitive and selective methods for its detection is essential.

Detection methods using different approaches have been developed for the in-place measurement of arsenic. Commercially available colorimetric tests are usually based on the highly sensitive and specific Gutzeit reaction, but their major disadvantage is that they require hazardous HgBr_2_ to be used^5,6^. As environmentally friendly alternatives, electrochemical detection methods can be combined with various sensing layers in affordable portable devices^7^. Direct electrical output of these devices is convenient to handle and analyze. Moreover, the detection of low concentration toxicants by electrochemical methods can be combined with various signal amplifier methods^8^ or microsystem technology on electrochemical nano-biochips^9,10^ enabling the construction of hand-held equipment for field monitoring with the required sensitivity.

Several attempts have been published for the electrochemical detection of arsenic deposited on biosensor surfaces^11^, including e.g. amperometry^9^, anodic stripping voltammetry (ASV)^12^ or cyclic voltammetry (CV)^12^. Engineered microorganisms and biological macromolecules immobilized on an electrode surface can provide a sufficiently sensitive sensing alternative for arsenic detection, often accompanied by appropriate selectivity. In the last two decades both cell-free (DNA- or protein-based) and cell-based sensing layers have been developed for the detection of inorganic and organic arsenicals in environmental samples^11,13–18^. Some of the whole-cell based sensors reach the maximum allowable concentration (MAC) limit and are highly selective, however, the time of response of cell-on-a-chip type sensors are still challenging^14^. Moreover, field-application of these sensors cannot meet practical requirements in general, due to their nature of being living organisms. On the other hand, most biomolecule containing sensing layers are capable to respond faster and have relatively good detection limits, but their stability under natural conditions is unsatisfactory, due to potential exposure to e.g. proteases. Although many efforts have been made to increase the stability of biomolecules or cells in sensors^19–21^, their prolonged storage and long-term use are still critical issues.

Although most protein-based arsenic sensing layers rely on enzyme inhibition^13^, for the direct detection of arsenic, metallothionein (MT), a protein known by its high metal binding capacity, has also been applied^22^. ArsR transcription factors are widely used as receptor molecules in whole-cell biosensors, however, in spite of its specific and strong As(III) binding ability, as far as we know the ArsR protein or its As-binding motif has not been used in biomolecular applications as sensing element. ArsR is an arsenic responsive transcriptional repressor protein from prokaryotes which controls the arsenic resistance genes organized in arsenic resistance (*ars*) operons^23^. Binding of As(III) at low concentrations to ArsR induces a conformational change in the protein allowing its release from DNA. Binding site for inorganic As(III) in ArsR is formed by three cysteines in all known repressor proteins. Some of the ArsR proteins from different organisms like ArsR from *Escherichia coli* (EcArsR) and *Acidithiobacillus ferrooxidans* (AfArsR) provide the three cysteine sidechains from the same monomer unit. Two of these are close to each other (CC or CXC, where X represents any amino acid except cysteine), while the third one coordinates from a slightly more distant position by marked bending of the polypeptide chain, enabling a strong binding and high selectivity for As(III) and Sb(III)^24^. Crystal structure of the arsenic bound form of AfArsR has been recently solved (PDB ID: 6J05)^25^, and confirms the earlier assumption that As(III) binds to the protein via the Cys95-Cys96-Cys102 side chains^26^. The same work suggests that the As(III) bond in EcArsR is similarly formed via the amino acids Cys32-Cys34-Cys37.

*Salmonella typhimurium* flagellin is a well-described protein capable of building long (up to 10 µm), highly stable filaments from thousands of monomer units by self-assembly^27^. Excellent polymerization ability of flagellin both in vivo and in vitro permits its production and purification in high amount to be an easy and cheap process. Flagellin has four domains: while D0 and D1 are deeply involved in filament formation, the variable D3 is not, and emerges on the surface of the filament. As it was demonstrated earlier, the D3 domain can be modified or even replaced by peptides or proteins without adversely influencing the polymerization ability^28–30^. In such a way, flagellin variants with variable features introduced into the D3 region can be used to build stable nanotubes carrying high functional site density on their surface enabling signal amplification. The length and composition of these nanotubes can be controlled and upon immobilization on sensor surfaces they can be potentially used as bioreceptor layers.

**Figure 1:**
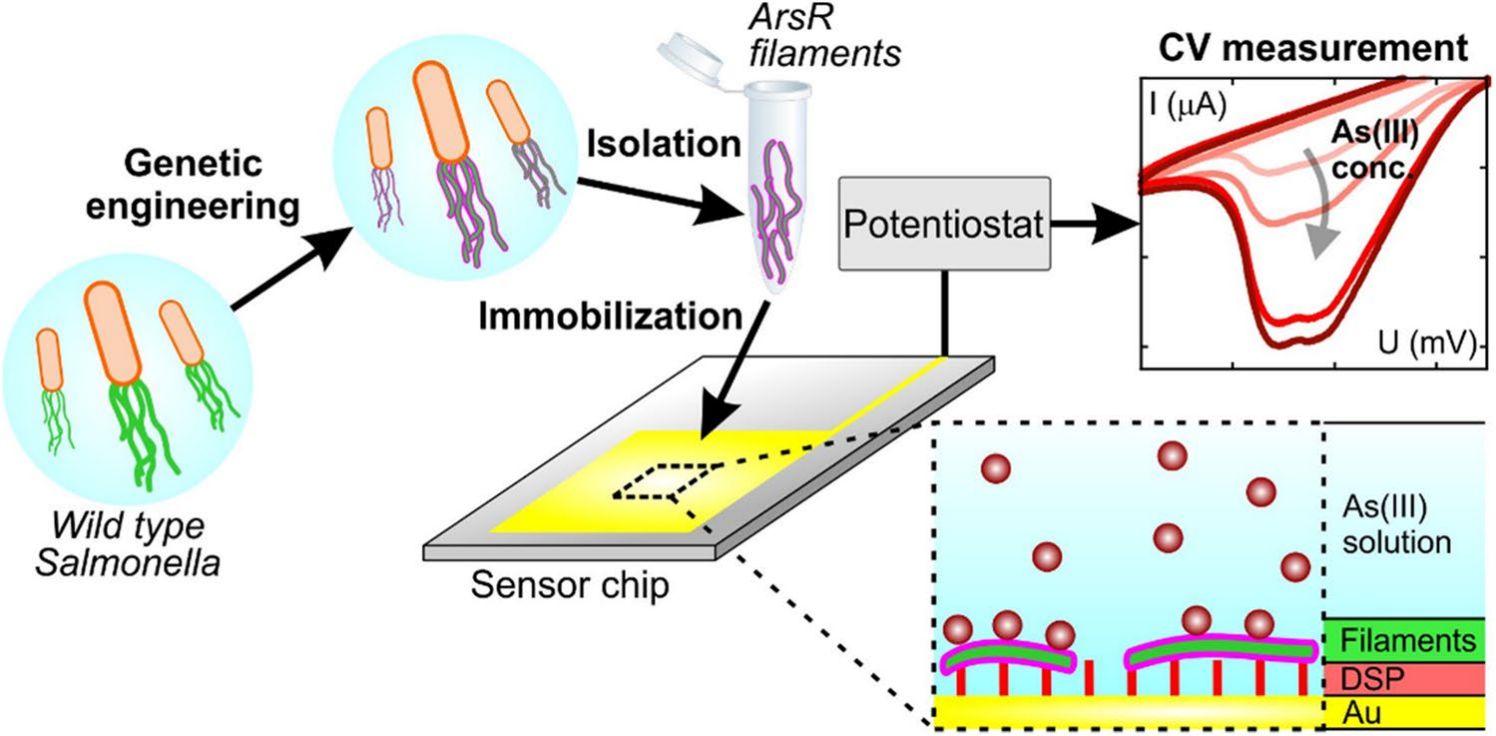
Schematic illustration of our As(III) detection methodology presented in this study. The workflow shows the production of genetically engineered flagellar filaments, their deposition on the gold working electrode surface as well as CV measurements on sample solutions containing As(III) at different concentrations.

In this research, we investigated whether the As-binding capacity of ArsR protein could be utilized to create a stable As-binding layer that could be easily fabricated, regenerated, and thus suitable for biosensor development. To achieve this goal, we prepared flagellin-based nanotubes with high surface binding site density, principally to detect As(III) concentration level in natural waters in the range of MAC. For this purpose, we chose the short arsenic binding polypeptide motifs from the transcriptional repressor ArsR proteins of *Escherichia coli* and *Acidithiobacillus ferrooxidans*. Built on our experience^28–30^ and available structural parameters for the ArsR proteins and wild type *Salmonella typhimurium* flagellin^31^, we designed and incorporated two different polypeptide motifs into flagellin and one of them in two different lengths, by replacing its D3 domain. Confirming both the polymerization and arsenic binding ability of each flagellin variant, we prepared filaments in vitro*,* immobilized covalently on electrode surfaces and detected their inorganic As(III) binding ability by CV (for a comprehensive scheme about our methodology, see Fig. 1). The sensing layers developed in our laboratory display further promising features, such as a short response time required to detect the electrochemical signal (i.e., within seconds), a partially regenerable sensing surface and, due to the stability of filament structure, the potential for long-term storage of the sensing layer-covered electrode.

## Results and discussion

### Design and gene construction of the arsenic binding flagellin variants

In this work we created flagellin-based proteins in which the D3 domain of *Salmonella typhimurium* flagellin was replaced by oligopeptides that are known to interact with arsenic. Since arsenic binding cysteines in both AfArsR (ArsR from *A. ferrooxidans*) and EcArsR (ArsR from *E. coli*) stem from the same protein monomer within a ten amino acid long polypeptide, however, they bear different cysteine arrangements (CXCX_2_C from *E. coli* and CCX_5_C from *A. ferrooxidans*), we aimed to incorporate both motifs into a flagellin scaffold by replacing its D3 domain. The replacement was performed using the pKOT-based vector described earlier, which contains the gene of the D3 deficient flagellin protein^1^. The DNA segments coding for the arsenic-binding oligopeptides were synthesized and ligated into the above plasmid between XhoI and SacI restriction sites.

Based on the proposed mechanism of As(III) binding to ArsR proteins, after coordination of the two adjacent cysteines to the metalloid, binding of the third one requires a significant bend in the peptide frame^26^. The distance between the N- and C-terminal ends of the D3 domain in wild type flagellin is about 6.7 Å (PDB ID: 1UCU). Based on the crystal structure of As-bound AfArsR, the conformational change upon As binding brings the two distant cysteines closer to each other, with the distance of about 7.9 Å (PDB ID: 6J05). Based on these parameters direct replacement of the flagellin D3 domain with the AfArsR Cys95-Cys102 segment may result in a somewhat tight structure upon As(III) binding weakening the interaction. In order to improve flexibility upon binding, we completed the binding motif of AfArsR by one amino acid on both sides (from the original sequences) and incorporated NCCHGTRDCA oligopeptide into flagellin (FliC-ArsR10). Moreover, linker dipeptides LE and EL from the translation of XhoI and SacI restriction sites, respectively, may further support the sufficient conformational change necessary for strong As(III) binding.

In the absence of a crystal structure, proposed folding of the unbound Cys32-Cys37 peptide in EcArsR, based on homology modelling, is less elongated compared to AfArsR, both due to the shorter polypeptide sequence (namely a hexapeptide) including all three binding cysteines and a more ordered (partially a helix) secondary structure (SWISS-MODEL ID: P37309). On the other hand, in EcArsR prior to the **C**V**C**DL**C** peptide, there is a highly conserved tripeptide, namely GEL, which may play role in the formation of the strong As-binding site. Considering all these conditions, from *E. coli* ArsR both the hexapeptide (**C**V**C**DL**C**) and the nonapeptide (GEL**C**V**C**DL**C**) motifs were incorporated into flagellin (FliC-ArsR6 and FliC-ArsR9). N- and C-terminal LE and EL dipeptides, respectively, ensure requisite flexibility for proper folding to strong As-binding.

### Protein expression and purification

Electroporated flagellin deficient SJW2536 *Salmonella* strain with plasmids encoding the flagellin variants (FliC-ArsR6, FliC-ArsR9 and FliC-ArsR10) were used for protein expression. The swimming ability of the mutant bacteria was studied by dark field optical microscopy and found the FliC-ArsR9 variant producing *Salmonella* to be motile, suggesting the formation of long surface filaments. This was confirmed by cell motility tests (Fig. S1A)^32^. Moreover, we observed a little motility for the FliC-ArsR6 variant producing *Salmonella*. TEM measurements revealed that only the FliC-ArsR9 variant forms long filaments on the bacterial surface (Fig. S1B), those are very short for the other two variants. On the other hand, we could see bundles of broken polymers in all samples. We quantified the ratio of expressed protein in the different fractions by SDS-PAGE (sodium dodecyl sulfate–polyacrylamide gel electrophoresis) as described previously^33^, which confirmed that after protein expression the FliC-ArsR9 variant is present in comparable amount on the cell surface and in polymeric form in the media, while the other two variants are mostly in broken filament form accumulating in the culture media (Fig. S1C). The proportion of these two fractions for each variant determines the most effective purification strategy. Expression and purification using the optimized protocol resulted in the yield of 55 mg protein/L culture for the FliC-ArsR9 variant, while for FliC-ArsR6 and FliC-ArsR10 the amount of pure protein was 10 and 17 mg/L culture, respectively.

### ITC investigation of the As-binding ability of monomeric FliC-ArsR variants

**Figure 2:**
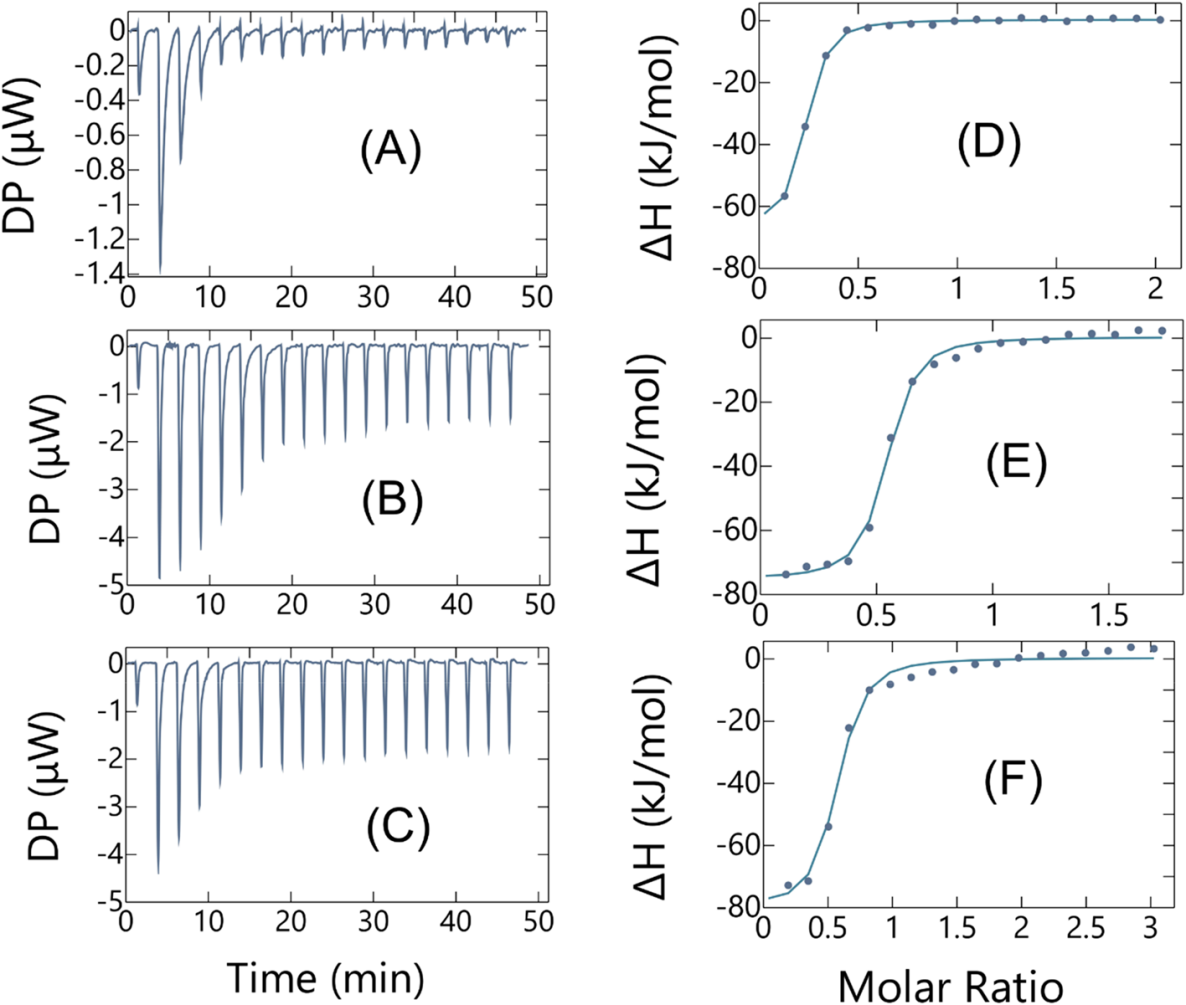
Isothermal calorimetric titration of FliC-ArsR variants with PAO at 25 °C. FliC-ArsR samples (c ~ 50 µM) were loaded into the cell and PAO (c ~ 0.5 mM) solution was injected in 2 μL portions. Titrations were performed in 100 mM HEPES (4-(2-hydroxyethyl)-1-piperazineethanesulfonic acid), 150 mM NaCl, 1 mM TCEP (pH 7.0) buffer. Beside the raw titration curves, changes in binding enthalpy (▪) of the injections are shown as a function of the molar PAO to FliC-ArsR ratio. The solid line is the least-squares fit to the data by using a one-binding-site model, resulting in the following parameters: (**A**,**D**) for FliC-ArsR9, stoichiometry N = 0.2, dissociation constant K_d_ = 6.9·10^−7^ M, binding enthalpy ΔH =  − 68.2 kJ/mol, (**B**,**E**) for FliC-ArsR10, N = 0.5, K_d_ = 3.7·10^−7^ M, ΔH =  − 76.2 kJ/mol, (**C**,**F**) for FliC-ArsR6, N = 0.5, K_d_ = 6.3·10^−7^ M, ΔH =  − 81.0 kJ/mol.

Arsenic binding ability of the purified flagellin variants was tested by ITC (isothermal titration calorimetry). This method measures the overall heat change arising from all reactions taking place after each titration step. The ITC data are presented as the baseline-adjusted raw data in the left panel and the peak-integrated, concentration-normalized heat of reaction versus PAO (phenylarsine oxide) to protein molar ratio in the right (Fig. 2). As we found, the heat change accompanying the side-reactions with dissolved As_2_O_3_ makes the evaluation of the titration curve too complicated, i.e. including the dehydroxylation of As(OH)_3_ followed by the formation of water molecules. For this reason, we decided to study the arsenic binding ability of the different monomeric FliC-ArsR variants by using PAO. Due to its phenyl group, the number of coordination sites in PAO is less, compared to As_2_O_3_, predicting a weaker binding site but could be suitable to demonstrate if a protein bears arsenic binding ability. In the measurements we applied 1 mM TCEP (Tris(2-carboxyethyl)phosphine hydrochloride) to avoid disulfide bond formation of cysteine sidechains. Buffer to protein and PAO to buffer background titrations were also carried out to exclude interactions between these components. Titration curves measured were fitted by the least squares method and evaluated by a one binding site model. As it can be seen from the titration curves, PAO interacts with each FliC-ArsR variant with submicromolar dissociation constant (Fig. 2) which are only a few times higher compared to the MAC limit (10 µg/L equivalent to 0.13 µM), indicating that at an appropriate and achievable protein concentration each variant could be suitable as sensing elements in biosensors. At the same time, the experimental (apparent/cumulative) equivalence point (N) was at about PAO to protein 1:2 molar ratio, which is rather unexpected taking into account that all ArsR variants contain 3 binding cysteines in suitable position, while PAO has only two binding sites. On the other hand, based on dynamic light scattering (DLS) measurements performed at different PAO to FliC-ArsR10 ratios under the same conditions the formation of a PAO to protein 1:2 complex can be excluded (Table S1). It means that the apparent N values in the ITC measurements stem from the shift that is due to other effects, i.e., conformational inhomogeneity which may lead to decreased binding functionality.

### Immobilization of polymeric arsenic binding flagellin variants on sensor surface

**Figure 3:**
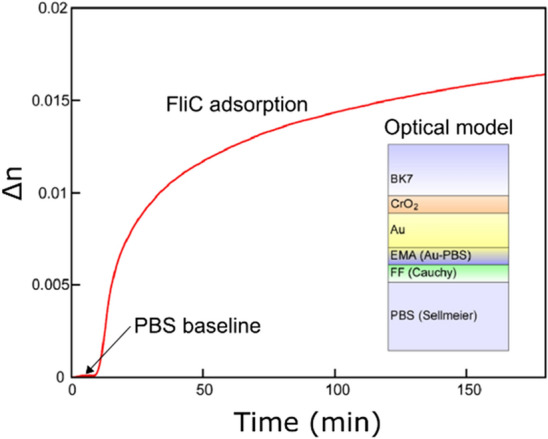
Change in the refractive index (Δn) near to the surface of a DSP-functionalized gold layer caused by the adsorption of flagellar filaments from their 0.5 mg/mL suspension. The kinetic curve was recorded by in situ spectroscopic ellipsometry. The inset shows the optical model used for data evaluation.

In vitro filament formation and thermal stability measurements carried out by circular dichroism (CD) spectropolarimeter indicated that these variants have high structural stability (Fig. S2) which makes them suitable to form stable nanotubes. In order to form a stable and dense layer of flagellar nanotubes on the gold surface, thiol-based covalent immobilization chemistry was applied. Best coverage was obtained using a DSP (dithiobis(succimidyl propionate)) concentration of 10 mg/mL and contact time of 90 min. The dynamics of the filament layer formation on a gold layer was monitored by in situ spectroscopic ellipsometry in the Kretschmann configuration, as shown in Fig. 3, using a Woollam M2000 rotating compensator spectroscopic ellipsometer (J. A. Woollam, Lincoln, Nebraska, US). In this setup, a glass slide covered by a 40 nm thick plasmonic gold layer was attached to a glass hemicylinder, and the gold-water interface was illuminated through this hemicylindrical lens, to utilize high-sensitivity plasmon-enhanced measurements. A ≈10 μL flow cell was attached to the gold-covered surface using an O-ring. Due to the hemicylindrical lens the angle of incidence can be changed between 45° and 70°, while the measured wavelength range is 400–1690 nm. The large amount of measured data made it possible to build a complex model shown in the inset of Fig. 3, including the thicknesses of all the films in the system: the CrO_2_ layer that enhances the sticking of the Au layer, the Au layer itself and its surface roughness modeled by the effective medium approximation (EMA (Au-PBS) in the inset). “FF” means the flagellar filament layer the dispersion of which was modeled using the Cauchy formula with two dispersion parameters. In Fig. 3 the change of the refractive index is plotted as a function of contact time, representing the number of filaments bound to the surface. In the initial phase of the immobilization process there is a quick change in the refractive index at the gold liquid interface, showing a quick build-up of the film. Then the formation of the nanotube layer slows down, after approximately an hour. The change in the refractive index of the total deposited filament amount shown in Fig. 3 corresponds to a surface mass density of approximately 200 ng/cm^2^.

**Figure 4:**
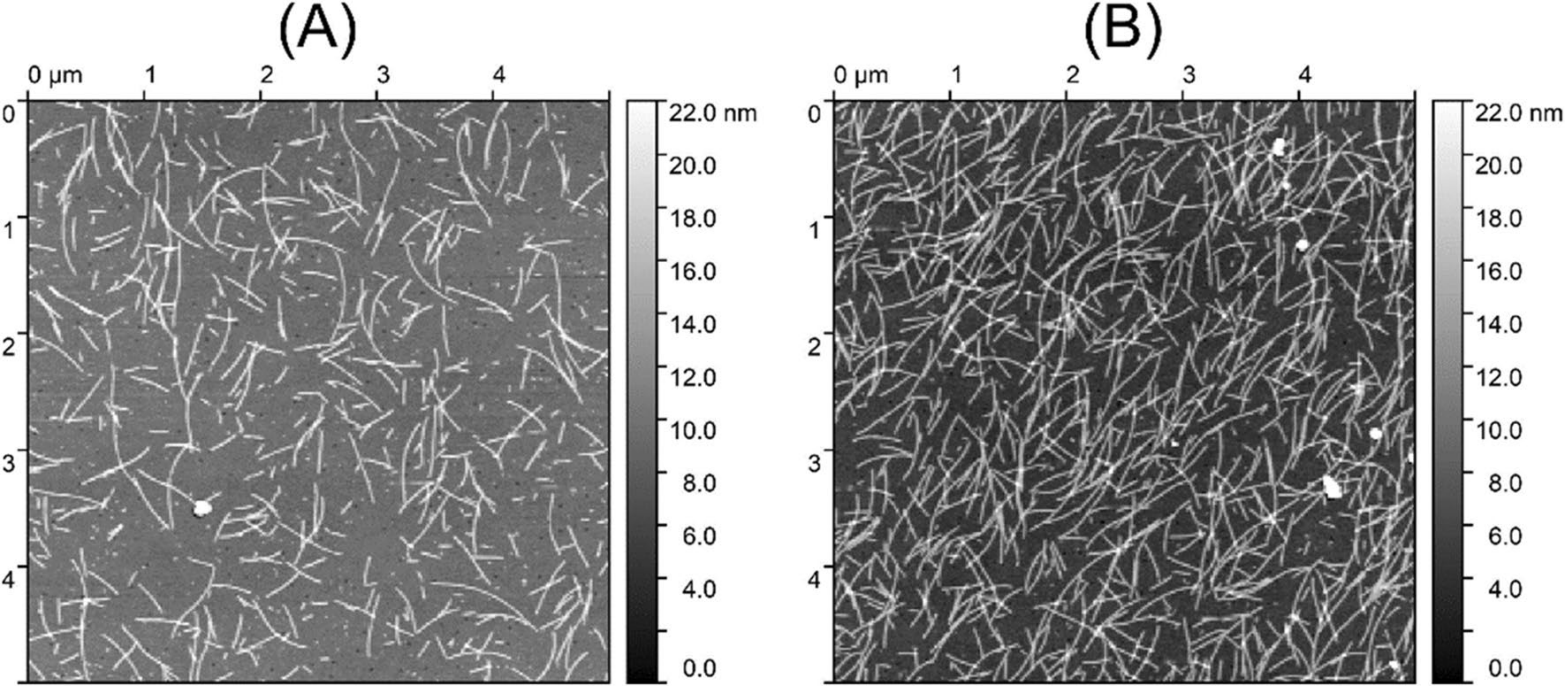
AFM images presented to compare the difference between the number of filaments bound to the gold surface without (**A**) and with (**B**) covalent bonds formed after activating the gold by DSP reagent. The vertical scale from the darkest to brightest points is 22 nm as shown in the color bar.

A comparison of the effect of activated surface to that without using DSP for the immobilization is shown in Fig. 4. A significantly better coverage for the covalent binding using DSP is revealed by atomic force microscopy (AFM) (AIST-NT SmartSPM 1000, AIST-NT Inc., Novato, California, US). As shown below, binding through DSP results in a stable layer which contributes to the good regenerability of the sensors.

### Electronic detection of As(III) using flagellar nanotube sensing layer made of FliC-ArsR9

Although arsenic is predominantly present in nature in the form of As(V), its direct electrochemical detection at neutral pH is considered to be almost impossible, due to the extreme stability and thus electrochemical inactivity of this oxidation state^34,35^. In contrast, electrochemical detection of As(III) is relatively simple at the same pH range. Several solutions have been developed for the measurement of As(V), e.g., acidifying the sample (down to pH 1)^36^, or reducing As (V) to As (III)^37^ before detection. In the case of protein-based biosensors only the latter method of sample pre-treatment could be applied.

**Figure 5:**
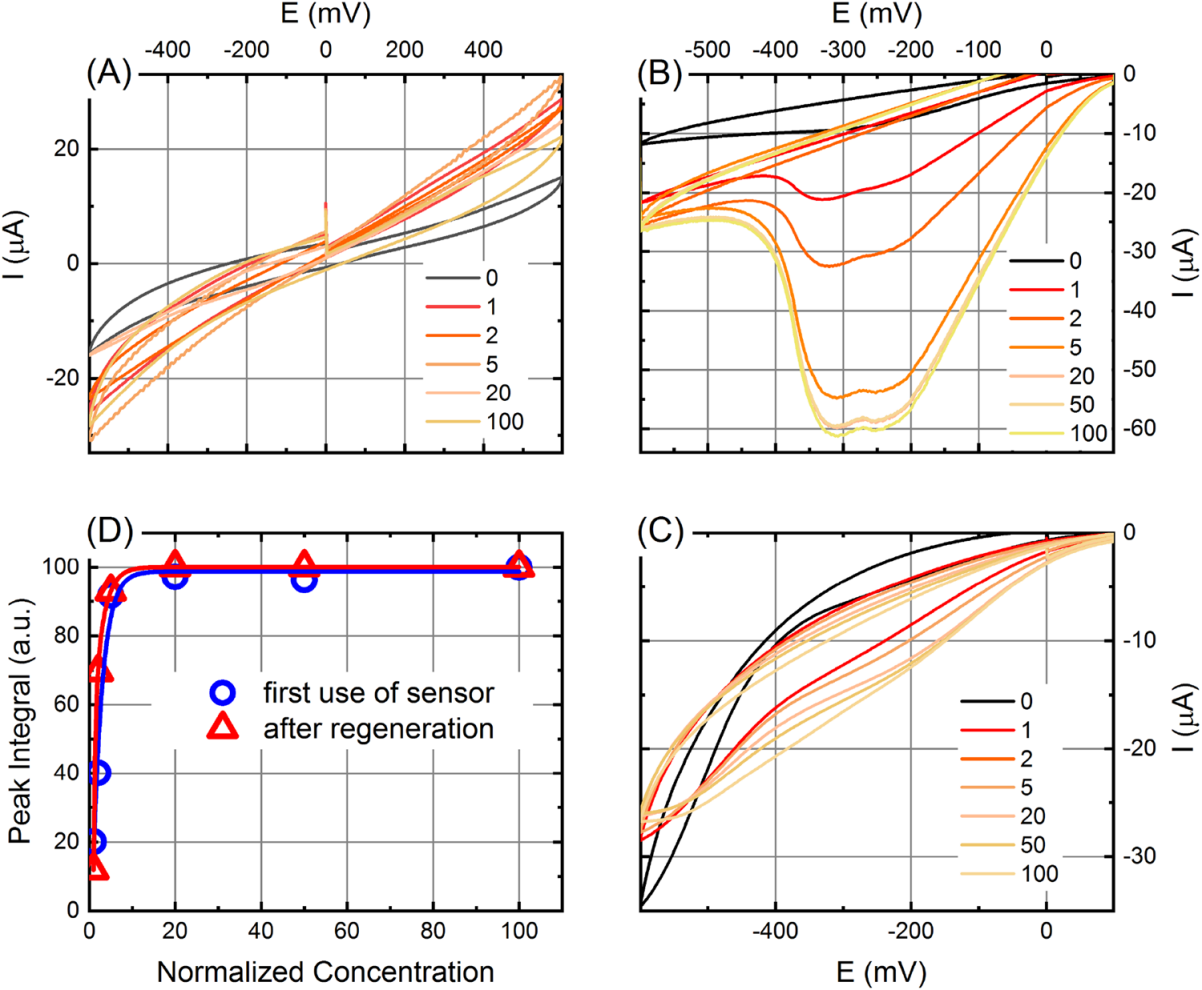
Set of CV curves measured in the presence of As(III) on wild type flagellar nanotubes (**A**) as well as on FliC-ArsR9 As-binding flagellar nanotubes before (**B**) and after (**C**) storage and regeneration. The numbers of the color code in the legend show the concentrations in units (U) of WHO threshold limit. Subfigure (**D**) shows the areas for FliC-ArsR9 filament cathodic peaks after first use and after regeneration calculated by averaging four measurements calculated using the method described in the text as a function of the As(III) concentration in U. The solid lines show exponential fits to the measured data points. The reproducibility of the peak integral values in graph D (determined by the repeated measurements) is smaller than the symbol size (typically a few percent), comparable to the width of the fitted lines.

In order to test the As(III) detecting capability of the CV setting in the presence of buffered As(III) solutions with different concentrations, we chose the nanotubes polymerized from the FliC-ArsR9 variant deposited on golden electrode. Hereinafter, arsenic concentration will be expressed in the units (U) of WHO threshold limit (1 U equal to 10 ppb or 0.13 µM)). As a negative control, a golden electrode covered by wild type flagellar nanotubes (FliC) was used in the 1–100 U As(III) concentration range. Redox reactions in the voltammograms can be interpreted as follows: first, the sample goes through the cathodic half-cycle up to + 0.6 V then back to Saturated Calomel Electrode (SCE) potential, where oxidation reactions may take place. Then the sample enters the anodic half-cycle where potential reduction reactions would result in a cathodic peak. All measurements were repeated at least three times. CV curves (Fig. 5) are represented using IUPAC conventions.

CV curves measured in As(III)-solutions at different concentrations using the wild type filament-covered golden electrode as a negative control clearly demonstrate that no redox reactions occurred nor in the anodic, neither in the cathodic region (Fig. 5A), as a proof that in absence of arsenic anchoring surface no signal can be detected.

Measurements performed using freshly prepared FliC-ArsR9 nanotube covered working electrode in As(III) containing solutions showed no anodic peak in the applied potential range (Fig. 5B), demonstrating that no As(III) to As(V) oxidation occurs. On the other hand, comparing the curve obtained from the measurement of As-free solution to other curves belonging to 1, 2, 5, 20, 50 and 100 U As(III), respectively, a cathodic peak appears in the measured potential range, and its intensity monotonously increases with the increasing As(III) concentration, up to saturation. This set of peaks can be associated with the cathodic reduction of the As(III) captured by specific binding to the FliC-ArsR9 nanotubes. Based on the shape of the envelope curves, it can be suggested that As(III) bound to the ArsR motif in an electronically rather complicated protein environment. Comparison of Fig. 5A,B proves that the effect is due to the presence of the As binding ArsR9 motifs on the surface of the working electrode. Moreover, significant and reproducible change of the CV profile even in 1 U As(III) solution (corresponds exactly to the MAC value) indicates that this sensing layer may provide an effective element of a biosensor for arsenic detection at low concentrations. This finding is well supported by our ITC measurements, where sub-micromolar dissociation constant for the FliC-ArsR9 – PAO interaction could be determined. The observed arsenic binding capability of the described setup allows the pre-selection of natural water samples for subsequent expensive and time-consuming analysis or, in combination with signal amplification techniques would allow the development of a sensor which can be applied directly for in-field testing.

In order to investigate the stability and reproducibility of the arsenic detection ability of the FliC-ArsR9 functionalized electrodes, the CV measurements were repeated after treatment in 10 mM dimercaptosuccinic acid (DMSA, Sigma Aldrich) solution followed by thorough rinse in DI water. DMSA is a strong chelating agent and widely used arsenic detoxifier and thus, it was intended to remove possible remaining As contamination from the chips. Functionalized electrodes after two months storage at 4 °C in PBS buffer or immediately after use were regenerated by DMSA for a few seconds or 5 min, respectively, prior to the subsequent measurement.

After storage and regeneration, the curves of the CV measurement series with FliC-ArsR9 coated electrodes clearly show that the cathode peak in the applied potential range is still present, although less pronounced than in the first measurement. The peak area starts to increase with increasing As(III) concentration (Fig. 5C), and then, a tendency towards saturation can also be observed for regenerated chips, suggesting that after the first use, the saturated As binding sites on the filaments were released by DMSA. We repeated the characterization process on freshly prepared sensor chips and with increased regeneration time of 5 min. A similar trend of increasing and then saturating cathodic peak area values were observed with the treatment and reuse of the chips several times in succession, however with decreasing maxima, indicating gradually decreasing As-binding ability. Therefore, this series of experiments confirms that flagellar nanotubes can be at least partially regenerated by treating the chip in DMSA solution at room temperature and retain their As (III) binding capacity for at least 2 months stored in buffer at 4 °C. This result also proves the outstanding stability of nanotubes built from flagellin and thus their suitability for the development of sensing layers. In addition, it is conceivable that by further refining the conditions, the recyclability of the layer can be further improved, which, given that there are very few examples of regenerable As-binding sensor layers in the literature, is a promising progress.

Evaluation of the cathodic peak area sets obtained from the measurements performed with freshly prepared and regenerated FliC-ArsR9 covered working electrodes is presented in Fig. 5D. The linear baseline of each CV curve was determined by linear interpolation of currents measured between 0 and -350 mV, and the peak area relative to this baseline was calculated. Peak areas were expressed as a percentage of the peak value for 100 U As(III) and plotted as a function of normalized As(III) concentration (Fig. 5D). Relative standard deviations (RSD) of the calculated peak areas for four parallel experiments were within a few percentages. The fitted exponential curves represent that as the As(III) concentration increases, the trend lines show saturation from about 20 U for both fresh and regenerated chips, despite the fact that the absolute peak intensities became smaller after regeneration. This saturation character also supports that the sensing process takes place on the protein, as the number of binding sites on the filament layer is limited. The presence of the As(III) concentration-sensitive range (0–20 U) suggests that this method can be used to determine not only the presence of arsenic in natural waters, but also to estimate its concentration in a wider range around the MAC value.

Specificity is also a very important attribute of a sensor layer. Although our present work did not cover the full investigation of various toxicants, especially heavy metal ions, the functionalized electrode we made was also tested in the presence of nickel ions. In this experiment, the chip was immersed in a solution containing 1 U As and Ni(II) ions at a 100-fold concentration. The shape and peak area of the CV curves measured in this way did not differ significantly from those measured in the arsenic-only solution (data not shown), from which it can be concluded that the detection of As contamination is not disturbed even by the presence of very high nickel excess.

## Conclusions

In this study, we present the design and fabrication of an As-sensitive sensing layer that can be mounted on the surface of a biosensor, the functional part of which is made of protein-based flagellar nanotubes. Flagellar nanotubes were constructed from mutant flagellins incorporating As-binding peptides by in vitro polymerization. Their stability similar to wild-type flagellin was confirmed by CD measurements and their strong arsenic binding ability by ITC measurements. Chips with golden surface were coated by the nanotubes in a covalent immobilization process using thiol-based surface chemistry. The arsenic detection ability of the chips thus prepared was tested by CV measurements in buffer solution, which showed that the sensitivity of the layer allows the detection of As (III) ions at concentrations as low as the MAC of water contamination. Moreover, up to 20 times the MAC, this setup may be suitable for more accurate determination of the concentration of arsenic. Based on the CV results obtained after DMSA treatment, it can be stated that the sensor layer is stable and partially regenerable, the platform developed for the detection of As (III) retains its As sensing ability. On the other hand, preliminary sensitivity test using Ni(II) ions showed that presence of this contaminant in high excess does not disturb arsenic detection, even at the MAC level.

Protein-based As sensor layers with one exception^22^ are reviewed and compared well^12,13^. Their sensitivity, like ours, often reaches the health limit, and some of them are pretty good in time-of-response, which is also true for the layer we developed. Data on specificity are reported in a few cases and only one of them contains a comprehensive study. Protein-based sensors, where reported, can usually be stored at 4 °C for several days. In a metallothionenin-based layer the protein was dried on filter paper, saturated with metal ions, and thus remained functional for months at room temperature^22^, however, it could only be used in this form after prolonged acidic treatment. Some sensor layers are formed on disposable electrodes, thus their regenerability is not relevant. In two cases where sensing was achieved using oxidase enzymes, the sensor could be completely regenerated several times by reversible redox reactions.

Based on the results presented in this work, the advantageous properties of the previously published nickel sensing layer^1^, and in comparison with other protein-based sensors created for arsenic detection, we can conclude that mutant flagellins containing toxic metalion binder peptide motifs, which are well designed and therefore retain their ability to polymerize, thereby capable to form stable nanotubes with high binding site densities, can be widely applicable as sensing layers for biosensors.

## Methods

### Cloning, expression and purification of As-binding flagellin variants

Complementary coding sequences of the different As-binding motifs (Table S2) were inserted into the gene of a D3-deleted variant of flagellin between the XhoI and SacI restriction sites of the pKOT-based plasmid as described earlier for the HG12 fusion variant^1^. Based on the result of in vivo filament formation experiments, expression of the fusion protein variants was carried out as described previously^33^, except that 25 mM dithiothreitol (DTT) (Promega, Madison, Wisconsin, US) was added to the purification buffer containing 20 mM tris(hydroxymethyl)aminomethane (Tris) (VWR International, Radnor, Pennsylvania, US), 150 mM NaCl (VWR) (pH 7.8) in order to avoid protein aggregation through intermolecular disulfide bond formation. Expressed protein was harvested by two strategies, considering the degree of the accumulation of polymeric protein in the culture media or on the cell surface for the different variants. Cell culture after overnight cultivation was centrifuged by 4300×*g* on a Multifuge 3 S-R (Heraeus, Hanau, Germany) for 30 min at 10 °C. The bacterial pellet was resuspended in 100 mL purification buffer and long filaments were removed from the cell surface by mechanical blending and separated from the cells and the buffer by two-step centrifugation: 10,000×*g* on a Biofuge primo R (Heraeus) for 30 min and 180,000×*g* on an Optima MAX-E Ultracentrifuge (Beckman Coulter, Brea, CA) for 35 min. Filamentous protein released to the culture media was collected by the addition of 2 (m/V)% polyethylene glycol (PEG6000) (Sigma Aldrich) and 1 (m/V)% NaCl, followed by a gentle shaking (50 rpm) at 20 °C for 40 min. Filaments were separated by centrifugation at 22,000×*g*, 15 min and washed twice by buffer. Polymeric protein samples from different sources were unified and purified using a standard protocol^33^, by the addition of 25 mM DTT to the purification buffer. Concentration of the pure monomeric protein solutions was determined in a DTT-free buffer using the extinction coefficient of ɛ(280 nm) = 10,430 M^−1^ cm^−1^ for all variants and the molecular weights of 43.03 kDa (FliC-ArsR6), 43.33 kDa (FliC-ArsR9) and 43.46 kDa (FliC-ArsR10). FliC-ArsR variants could be stored for several months in polymeric form, suspended in buffer at 4 °C. Before further measurements, flagellin variants were thoroughly washed with DTT-free buffer.

### In vivo filament formation of different As-binding flagellin variants

In vivo filament formation capability of the different As-binding flagellin variants, namely FliC-ArsR6, FliC-ArsR9 and FliC-ArsR10, was investigated by cell motility tests^32^. Protein expression and the emergence of monomeric or polymeric forms on the cell surface and in the culture media, were studied by sodium dodecyl sulfate polyacrylamide gel electrophoresis (SDS-PAGE) after various treatments, such as ultracentrifugation, heat treatment or trichloroacetic acid (TCA) precipitation. Selected cells were visualized by dark-field optical microscopy on an Olympus BX50 microscope (Shinjuku, Tokyo, Japan) and transmission electron microscopy (TEM) on a FEI Talos F200X G2 instrument with X-FEG field emission gun, operated at 200 kV accelerating voltage, visualizing the results on bright-field images. Samples were deposited onto Cu TEM grids covered by a Formvar/carbon film and negatively stained with 2% phosphotungstate solution prepared from the acidic form (PTA, pH 7.0) (Sigma Aldrich, St. Louis, Missouri, US) to enhance image contrast.

### Isothermal titration calorimetry

ITC experiments were carried out at 25 ± 0.2 °C by a MicroCal PEAQ-ITC calorimeter. Arsenic binding flagellin variants in monomeric form were prepared in 100 mM 4-(2-hydroxyethyl)-1-piperazineethanesulfonic acid (HEPES) ((Sigma Aldrich), 150 mM NaCl, 1 mM Tris(2-carboxyethyl)phosphine hydrochloride (TCEP) (Sigma Aldrich) (pH 7.0). Before the measurement 10% v/v DMSO was added to the solution. As a titrant, phenylarsine oxide (PAO) (Sigma Aldrich) was first dissolved in pure dimethyl sulfoxide (DMSO) (Sigma Aldrich) to an appropriate concentration then diluted 10 times with 100 mM HEPES, 150 mM NaCl (pH 7.0) buffer containing 1 mM TCEP. A typical experiment consisted of a ~ 0.5 mM PAO solution titrated into a ~ 5 µM protein solution in the same buffer. Interaction between PAO and the buffer or the buffer and protein was checked by PAO-to-buffer and buffer-to-protein titrations, respectively. The ITC data were fitted to a one-binding-site model with the MicroCal PEAQ-ITC Analysis Software package provided by MicroCal, using non-linear least-squares algorithm.

### Construction and immobilization of flagellar nanotubes

Buffered solutions of the purified monomeric flagellin variants were induced by the addition of ammonium sulfate (AS) ((Scharlab, Barcelona, Spain) solution to a final concentration of 0.8 M to form filaments in vitro^38^. Polymerized protein was resuspended in phosphate-buffered saline (PBS) (Invitrogen) with 1 mM TCEP and the filaments were monomerized by heat treatment (70 °C, 10 min). After centrifugation at 300,000×*g* on an Optima MAX-E Ultracentrifuge (Beckman Coulter) for 20 min, protein solution of c.a. 2 mg/mL was re-polymerized by the addition of AS, to a final concentration of 0.6 M for at least 3 h incubation at room temperature (RT). Size distribution of reconstructed filaments was estimated by dynamic light scattering (DLS) using a Zetasizer Nano-ZS (Malvern Panalytical, Malvern, UK) instrument. A typical average length of 200–300 nm was obtained.

The As-binding ability of engineered polymeric flagellin variants was tested by immobilizing the nanotubes on Si wafer integrated sensor chips. The as-prepared clean gold electrodes of the sensor chips were first activated by dithiobis(succimidyl propionate) (DSP, Sigma-Aldrich) and then covalently functionalized with the As-binding or wide-type filaments through the thiol groups of the crosslinker^1^. The chips were stored in PBS at 4 °C.

### Cyclic voltammetry

CV measurements were performed as described earlier^1^. As_2_O_3_ (Sigma Aldrich) was dissolved in distilled water (typically by stirring for several days) and filtered. Arsenic stock solution was diluted first to an appropriate concentration then subsequent increasing amounts of As(III) were added to 100 mM HEPES, 150 mM NaCl (pH 7.0) buffer containing 1 mM TCEP. After testing the CV characteristics in As(III)-free solution, the effect of As(III) concentration was studied by the addition of variable amounts of As(OH)_3_ solution into the measurement cell.

## Supplementary Information

Supplementary Information
