# Supplementary material for: Flagellin-based electrochemical sensing layer for arsenic detection in water
Source: Sci Rep. 2021 Feb 10;11:3497. doi: 10.1038/s41598-021-83053-y (PMC7876115; doi:10.1038/s41598-021-83053-y)
Supplement: Supplementary file 1 — Supplementary Information [file 41598_2021_83053_MOESM1_ESM.pdf]

Supplementary Material

Flagellin-based electrochemical sensing layer for arsenic detection in water

Hajnalka Jankovics[a,#], Patrik Szekér[a], Éva Tóth[a], Balázs Kakasi[a], Zoltán Lábadi[b], András Saftics[b], Benjamin Kalas[b], Miklós Fried[b,c], Péter Petrik[b], Ferenc Vonderviszt[a,b]

[a] Research Institute of Biomolecular and Chemical Engineering, University of Pannonia, P.O. Box 125, Veszprém, 8200, Hungary

[b] Institute of Technical Physics and Materials Science, Centre for Energy Research, P.O. Box 49, Budapest, 1525, Hungary

[c] Institute of Microelectronics and Technology, Óbuda University, P.O. Box 112, Budapest, 1431, Hungary

[#] Corresponding author (e-mail: jankovicsh@almos.uni-pannon.hu)

In vivo filament formation of different As-binding flagellin variants

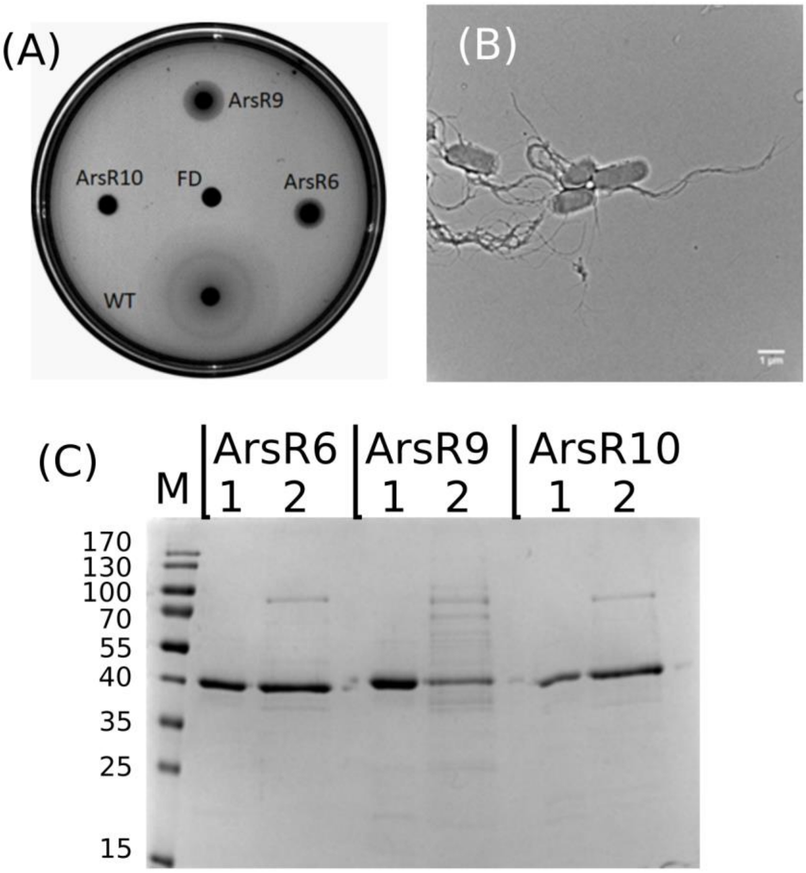

Figure S1. FliC-ArsR9 expression, (A) Motility plate (WT: wild type flagellin; FD: flagellin deficient; ArsR6, ArsR9, ArsR10: FliC-ArsR6, FliC-ArsR9 and FliC-ArsR10 producing *Salmonella*, respectively), (B) TEM image of cells and released filament bundle of FliCArsR9, (C) SDS PAGE, filament accumulation on the cell surface (1) and culture media (2), for FliC-ArsR6, ArsR9 and ArsR10, respectively. (M: PageRuler Prestained Protein Ladder (Thermo Fisher Scientific), MW given in kDa)

Dynamic light scattering (DLS) measurements

The thermally treated and filtered solutions of FliC-ArsR variants mixed with PAO at different PAO to protein ratios, incubated for an hour or overnight, were all analyzed by dynamic light scattering (DLS) using a Zetasizer Nano ZS instrument (Malvern Instruments, Malvern, UK) at 25 °C. Samples were measured under identical buffered conditions (100 mM HEPES, 150 mM NaCl (pH 7.00) containing 1 mM TCEP using 120 µL disposable polystyrene cuvettes. For each

sample, three parallel size measurements were carried out and the mean of the three measurements was calculated. Data were analyzed using the DTS (Version 5.02) software supplied by Malvern.

To figure out whether PAO can really bind two protein molecules forming a homodimer (that can be characterized by the N value of the ITC experiment), we performed DLS measurements under the same conditions, but at different PAO to FliC-ArsR10 ratios, namely 1:1, 0:1, 1:2, 2:1, 1:0. As a control, the size distribution of monomeric wild type FliC was also measured. Size distribution curves from DLS experiments for all M:L ratios (obviously containing protein) demonstrated the presence of exclusively monomeric species, with a size distribution maximum very similar to wild type monomeric flagellin, accordingly (Table S2). It means that the apparent N values in the ITC measurements stem from the shift that is due to other effects, i.e., conformational inhomogeneity which may lead to decreased binding functionality.

**FliC-ArsR10 to PAO molar ratio**	**Average particle size by intensity (nm)**
1:0	13.4 ± 3.9
1:1	13.7 ± 3.9
2:1	11.9 ± 3.5
1:2	11.8 ± 3.4
monomeric w.t. flagellin	12.3 ± 3.6

Table S1: Size distribution of FliC-ArsR10 – PAO species formed at different protein to PAO molar ratios under the conditions equal to that applied in the ITC titrations (100 mM HEPES, 150 mM NaCl, 10% DMSO, pH 7.0), measured by DLS after 1 hour or overnight incubation, compared to the size distribution of monomeric wild type flagellin (same conditions).

Circular dichroism (CD) for thermal stability measurements

The temperature associated depolymerization and unfolding of filamentous FliC-ArsR variants were measured by a Jasco J-1100 CD spectropolarimeter. The measurements were performed

in 1 mg/mL protein solution dissolved in the protein purification buffer, containing 20 mM Tris, 150 mM NaCl (pH 7.8) containing 1 mM TCEP in a Peltier thermostatted (PTC-514) cell holder using 1 mm rectangular quartz cuvette. The unfolding was monitored at 222 nm between 20 - 70 °C applying 1 °C/min heating rate and 0.1 °C increments. The thermal denaturation temperature values were determined based on the first derivatives of the thermal unfolding stability curves.

Thermal stability of the polymeric form of new flagellin variants was measured and compared to wild type flagellin using CD measurements. Each FliC-ArsR variants has a melting point (T_m) around 50 °C, close to that obtained for wild type flagellin (49.4 °C), but even a bit higher (Fig. S2). These results indicate that these variants have high structural stability which makes them suitable for sensor applications.

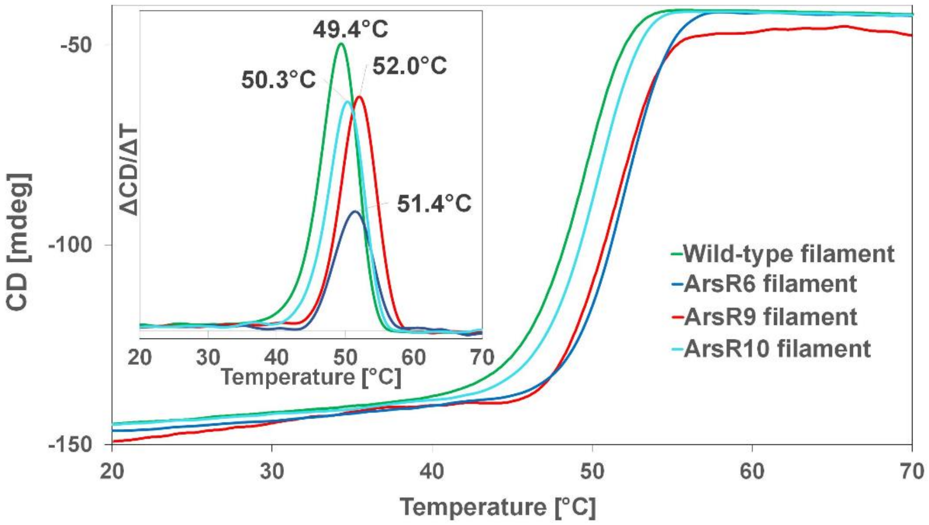

Figure S2. Normalized thermal stability circular dichroism curves of the different FliC-ArsR (noted as ArsR) variants compared to wild-type flagellin between 20-70 °C applying 1 °C/min heating rate at 222nm. The first derivative curves indicate the exact melting temperatures in 20 mM Tris, 150 mM NaCl (pH 7. 8) containing 1 mM TCEP.

DNA oligos for the preparation of the coding sequence

Motif	forward (5'-..-3')	reverse (5'-..-3')

ArsR10	tcgagAACTGCTGCCATGGCACCCGCGATTGCGCGgagct	cCGCGCAATCGCGGGTGCCATGGCAGCAGTTc
ArsR9	tcgagGGCGAACTGTGCGTGTGCGATCTGTGCgagct	cGCACAGATCGCACACGCACAGTTCGCCc
ArsR6	tcgagTGCGTGTGCGATCTGTGCgagct	cGCACAGATCGCACACGCAc

Table S2: Coding sequence of DNA oligos for the preparation of different As-binding flagellin variants by oligo annealing. Uppercase: arsenic binding motif coding sequence, lowercase: digested-like restriction enzyme cleavage site overhangs.
